# Vaginal laparoscopically assisted radical trachelectomy in cervical clear cell adenocarcinoma

**DOI:** 10.3332/ecancer.2013.373

**Published:** 2013-11-14

**Authors:** Sara Iacoponi, Maria Dolores Diestro, Ignacio Zapardiel, María Serrano, Javier De Santiago

**Affiliations:** Gynaecological Oncology Unit, La Paz University Hospital, Madrid, Spain

**Keywords:** clear cell carcinoma, radical trachelectomy, fertility sparing, cervical adenocarcinoma

## Abstract

Adenocarcinoma of the cervix is a rare condition that has shown an increase in incidence, especially in the 20- to 34-year-old group. Adenocarcinoma represents about 5–10% of all tumours in this area, and, among these, the clear cell type accounts for 4–9%. This type of tumour affects mainly postmenopausal women but also occurs in young women with a history of prenatal exposure to diethylstilbestrol (DES). The prognosis for adenocarcinoma of the cervix is poor overall and worse for the clear cell variety. This article discusses a case of clear cell adenocarcinoma of the cervix, unrelated to intrauterine exposure to DES, in a woman of childbearing age who wished to preserve her fertility and was therefore treated by radical vaginal trachelectomy and pelvic lymphadenectomy.

## Introduction

Worldwide, cervical cancer has an annual incidence of 493,000 cases [1]. Of the women diagnosed with cancer of the cervix, 43% are under 45 years of age. The incidence in women aged 20–49 years is 1.5-14.9 per 100,000 [2–4].

Adenocarcinoma of the uterine cervix is a rare type of tumour, although the incidence of these tumours has increased in recent years, particularly in the 20- to 34-year-old age group [5]. Adenocarcinoma represents around 5–10% of all tumours in this location, and, among all of these, clear cell adenocarcinoma accounts for 4–9% [6]. This type of tumour affects mainly postmenopausal women, although it also occurs in young women with a history of prenatal exposure to diethylstilbestrol (DES). The prognosis for adenocarcinoma of the cervix is poor overall and somewhat worse for the clear cell variety. We present a case of clear cell adenocarcinoma of the cervix, unrelated to intrauterine exposure to DES, in a woman of childbearing age who wished to preserve her fertility and was therefore treated with radical vaginal trachelectomy (RVT).

## Clinical case

A 28-year-old woman, nulligravida, presented at our cervical disease clinic with a suspected low-grade squamous intraepithelial lesion (LGSIL) and human papilloma virus (HPV), positive cytology (carried out at another centre), and bleeding during intercourse for the last year. She had no substance abuse or significant medical or family history.

**Figure 1.**
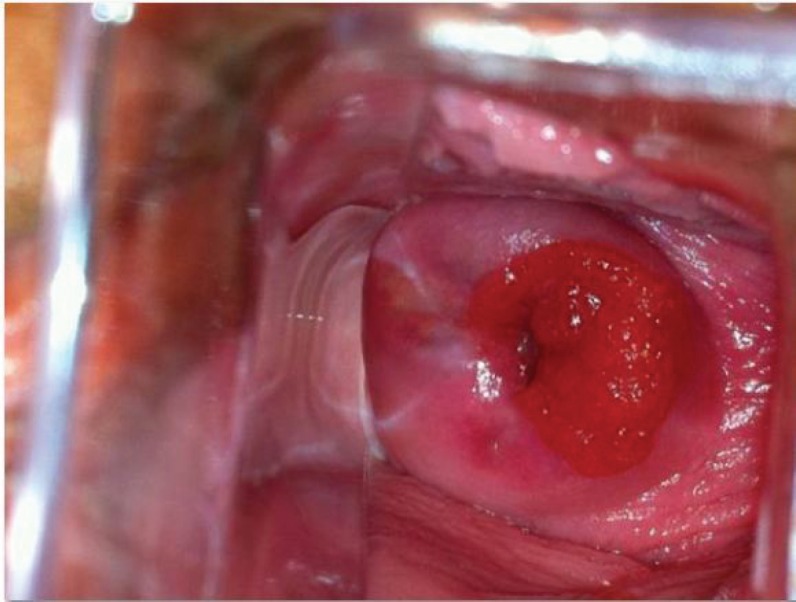
Tumour outgrowth, 2 cm in diameter.

**Figure 2.**
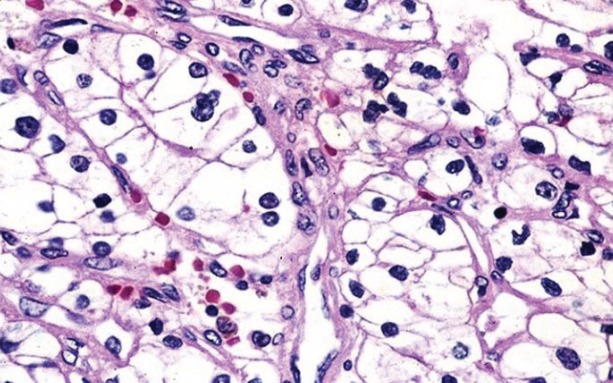
A histological investigation of clear cell adenocarcinoma.

A 2-cm vascularised tumour outgrowth, indurated and bleeding, was found on investigation (Figure 1); colposcopy revealed an area of type 1 changes [7], with a vascularised lesion approximately 2 cm in diameter, extending from the cervical canal to the posterior labium; this was biopsied. A histopathological analysis of the specimen showed it to be a clear cell adenocarcinoma (Figure 2) with a Ki67 index of 60%, low-to-moderate nuclear immunoreactivity for p53 and cytokeratin 7, and negative hormone receptors.

**Figure 3.**
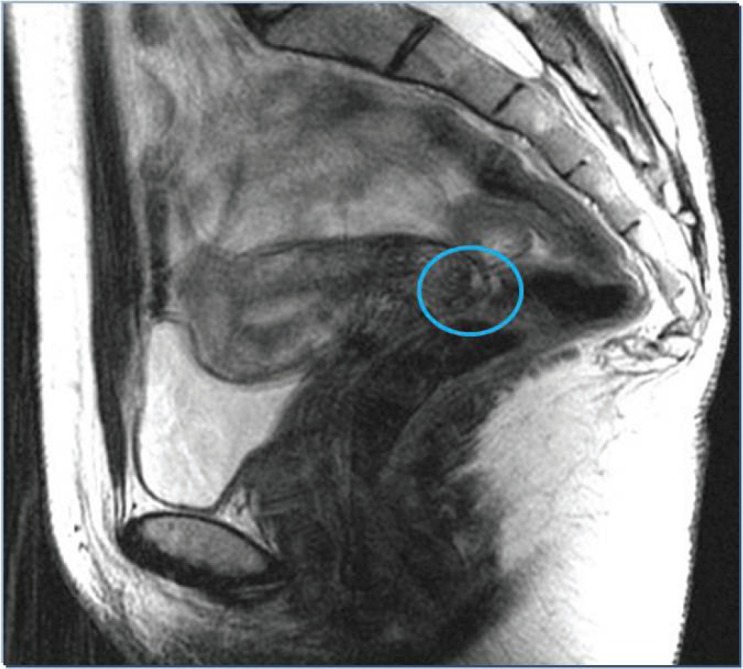
MRI: 25-mm cervical lesion with no infiltration into the parametria.

Following this, a further investigation was carried out on the patient, including gynaecological examination, gynaecological ultrasound, laboratory tests, and tumour markers, which were all normal. Magnetic resonance imaging (MRI) was also carried out and revealed a cervical lesion 25 mm in diameter with no infiltration into the parametria (Figure 3).

A diagnostic cone biopsy was performed; the histopathological result was a clear cell adenocarcinoma measuring 20 x 15 mm with 3-mm stromal infiltration, clear margins, and no lymphovascular invasion. The tumour stage was determined to be International Federation of Gynecology and Obstetrics (FIGO) IB1; since the patient wanted to preserve her fertility, she was offered a radical trachelectomy with selective biopsy of the sentinel lymph node (SLN).

**Figure 4.**
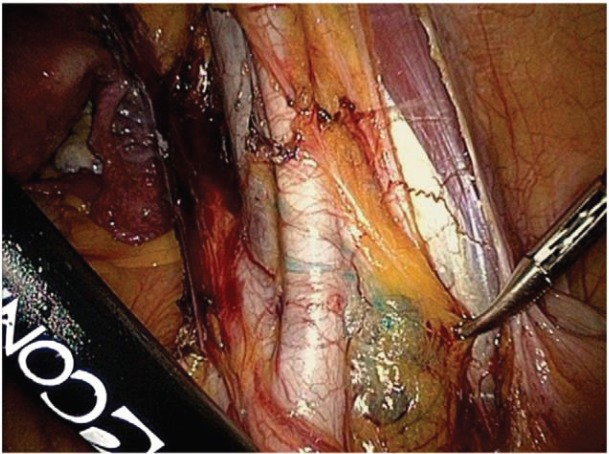
Identification of SLN in the area of the right external iliac vessels.

The SLN technique included injection at the four cardinal points of the cervix, on the day before surgery, of Tc-99m (10 MBq in 0.2 mL of saline solution in each injection) and lymphoscintigraphy prior to surgery. Subsequently, in the operating theatre, 4 mL of undiluted methylene blue was injected into the same points (1 mL at each point), and one lymph node was identified in the external iliac vessels on either side. These were removed laparoscopically; intraoperative analysis found them to be negative (Figure 4).

**Figure 5.**
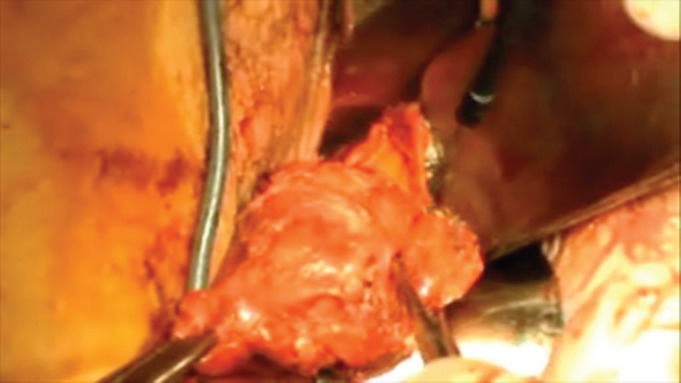
The surgical specimen including the cervix and parametria following the RVT.

Since our centre is involved in a validation study on SLNs in cervical cancer, a bilateral laparascopic pelvic lymphadenectomy was then performed. Next, an RVT was carried out, with no complications (Figure 5). The final histopathological analysis showed no residual tumour; 17 pelvic lymph nodes were identified, with no metastases.

Three months later, the patient was symptom free; an MRI showed a 3-cm lymphocoele in the left groin, which had abated at the next review.

The patient was followed up quarterly at the gynaecological oncology clinic, with a gynaecological examination, vulvoscopy, colposcopy, and laboratory tests; at six months after the RVT, the gynaecological ultrasound and MRI showed no further effects. After 18 months of follow-up, the patient is still symptom free and is planning a pregnancy.

## Discussion

Cervical cancer is the second most common cancer in Europe in women of childbearing age. More than 40% of stage I patients are under 40 years of age. There is a documented trend toward delaying motherhood to the late 30s and early 40s, leading to a higher proportion and more patients being diagnosed with cervical cancer before having started or completed their family. The review by Leitao demonstrates that a very high proportion of patients with gynaecological cancer are under 40 years of age [4].

This intervention includes amputation of the cervix below the uterine isthmus, with removal of parametrial tissue and bilateral pelvic lymphadenectomy. In selected cases, it may be decided to carry out a biopsy of the SLN, as in our case.

The selection criteria for radical trachelectomy (with some variability from one author to another) are a strong desire for pregnancy, confirmed diagnosis of cervical cancer (any histological type except for neuroendocrine tumour and sarcoma), tumour size equal to or less than 2 cm, less than 50% stromal invasion, FIGO stage IA1 with LVI, IA2 or IB1, absence of any evidence of metastasis in or away from the pelvic lymph nodes, and age under 40 years [4, 8–10].

Our clinical case met the inclusion criteria, as the patient was a woman of childbearing age who had a strong desire to start a family, with stage IB1 clear cell adenocarcinoma; therefore, radical trachelectomy would be the technique of choice.

More than 900 cases have been published in the literature, all performed at a small number of specialist centres. The recurrence rate and mortality following this procedure are 4.2% and 2.8%, respectively, which are similar to those offered by radical hysterectomy without fertility sparing [8].

The SLN technique would allow pelvic lymphadenectomy to be avoided, thus reducing complications and morbidity [11].

The most important factors for disease prognosis are the tumour stage, size, growth pattern, nuclear atypia, and mitotic activity. In the case of clear cell carcinoma at stages I–II, five-year survival does not exceed 60%, when considering this subtype of poor prognosis [12].

It is not completely clear whether clear cell cervical adenocarcinoma has a worse prognosis than squamous cell cancer, and therefore in our case, we decided not to exclude the patient for a fertility-sparing technique [13–15].

Korhonen suggested, having analysed 163 cases of primary cervical adenocarcinoma of different subtypes, that the prognosis for clear cell and non-clear cell adenocarcinoma is similar [14].

However, in a review of the literature, Niibe suggests that the five-year survival rate in cervical adenocarcinoma is worse than that in squamous cell cancer [16]. All of the authors agree that the most important of the principal factors influencing the prognosis in each histological type of cancer of the uterine cervix is the disease stage [13, 15, 16].

In this case, the FIGO tumour staging was IB1, and the standard treatment for these initial stages is radical hysterectomy together with pelvic lymphadenectomy, but in view of the patient’s young age, it seemed reasonable to attempt fertility-sparing treatment.

When the selection criteria are not met, another fertility-sparing option is neoadjuvant chemotherapy followed by conisation; this technique was first proposed by Landoni *et al* in 2007 (17). In his work, Landoni analyses his experience from 1995 to 2007, in a total of 21 patients. Twenty-one patients aged < 40 years, with tumours measuring < 3 cm, received three cycles of chemotherapy with cisplatin 75 mg/m^2^, paclitaxel 175 mg/m^2^, and ifosfamide 5 g/m^2^, followed by conisation and pelvic lymphadenectomy.

After 69 months of follow-up, the authors observed no recurrence, and there were nine pregnancies; they therefore concluded that neoadjuvant chemotherapy could reduce tumour volume and allow safe removal of the cervical cone [17].

There are few references in the literature to obstetric results after radical trachelectomy. In his review, Boss analysed a total of 355 patients on whom he performed RVT, observing that only 43% wished to become pregnant and, of these, 70% were successful. Losses in the first trimester were 21%, comparable with that of the general population, but losses in the second trimester were 8%, almost double that of the general population, with 42% full-term pregnancies [18].

To prevent the obstetric complications associated mainly with radical trachelectomy, some authors such as Shepherd and Plante suggest waiting for six months to one year before trying to get pregnant [19, 20]. Other authors suggest bi-weekly follow-up between 18 and 28 weeks, then weekly visits including measurement of cervical length at every visit [21].

In conclusion, treatment for women with early-stage cervical cancer can include fertility sparing, always on a personalised basis. Further studies are needed on controversial histological types such as clear cell adenocarcinoma, but the final decision must always be given to the correctly informed patient.

## Conflicts of interest

The authors declare that they do not have any financial or potential conflicts of interest of any kind.
